# 4D perfusion CT of prostate cancer for image-guided radiotherapy planning: A proof of concept study

**DOI:** 10.1371/journal.pone.0225673

**Published:** 2019-12-19

**Authors:** Lucian Beer, Stephan H. Polanec, Pascal A. T. Baltzer, Georg Schatzl, Dietmar Georg, Christian Schestak, Anja Dutschke, Harald Herrmann, Peter Mazal, Alexander K. Brendel, Shahrokh F. Shariat, Helmut Ringl, Thomas H. Helbich, Paul Apfaltrer

**Affiliations:** 1 Department of Biomedical Imaging and Image-guided Therapy, Medical University of Vienna, Vienna, Austria; 2 Department of Radiology and Cancer Research UK Cambridge Center, Cambridge, United Kingdom; 3 Department of Urology, Medical University of Vienna, Vienna, Austria; 4 Department of Radiation Oncology, Medical University of Vienna, Vienna, Austria; 5 Christian Doppler Laboratory for Medical Radiation Research for Radiation Oncology, Medical University Vienna, Austria; 6 Clinical Institute of Pathology, Medical University of Vienna, Vienna, Austria; 7 Department of Neuroradiology, University Medical Center Mannheim, Medical Faculty Mannheim, University of Heidelberg, Mannheim, Germany; University of Nebraska Medical Center, UNITED STATES

## Abstract

**Purpose:**

Advanced forms of prostate cancer (PCa) radiotherapy with either external beam therapy or brachytherapy delivery techniques aim for a focal boost and thus require accurate lesion localization and lesion segmentation for subsequent treatment planning. This study prospectively evaluated dynamic contrast-enhanced computed tomography (DCE-CT) for the detection of prostate cancer lesions in the peripheral zone (PZ) using qualitative and quantitative image analysis compared to multiparametric magnet resonance imaging (mpMRI) of the prostate.

**Methods:**

With local ethics committee approval, 14 patients (mean age, 67 years; range, 57–78 years; PSA, mean 8.1 ng/ml; range, 3.5–26.0) underwent DCE-CT, as well as mpMRI of the prostate, including standard T2, diffusion-weighted imaging (DWI), and DCE-MRI sequences followed by transrectal in-bore MRI-guided prostate biopsy. Maximum intensity projections (MIP) and DCE-CT perfusion parameters (CTP) were compared between healthy and malignant tissue. Two radiologists independently rated image quality and the tumor lesion delineation quality of PCa using a five-point ordinal scale. MIP and CTP were compared using visual grading characteristics (VGC) and receiver operating characteristics (ROC)/area under the curve (AUC) analysis.

**Results:**

The PCa detection rate ranged between 57 to 79% for the two readers for DCE-CT and was 92% for DCE-MRI. DCE-CT perfusion parameters in PCa tissue in the PZ were significantly different compared to regular prostate tissue and benign lesions. Image quality and lesion visibility were comparable between DCE-CT and DCE-MRI (VGC: AUC 0.612 and 0.651, p>0.05).

**Conclusion:**

Our preliminary results suggest that it is feasible to use DCE-CT for identification and visualization, and subsequent segmentation for focal radiotherapy approaches to PCa.

## Introduction

Radiotherapy encompassing external beam intensity-modulated radiation therapy (IMRT) and brachytherapy represent established treatment options for patients with localized prostate cancer (PCa). In the current daily clinical routine, it is still recommended to include the whole prostate in the clinical target volume for radiotherapy [1,2]. As local recurrences mainly occur at the site of the primary tumor lesion [3], ongoing efforts are being made to establish a boost focused on the tumor tissue, with a current standard dose for the remaining prostate [4]. Based on computerized treatment plan optimization algorithms and respective IMRT-based dose delivery techniques, even multiple clinical target volumes (CTV) with different dose prescriptions can be treated within one treatment session, and, at the same time, reducing the dose to organs at risk (OAR) [5–8].

However, one of the prerequisites for focal radiotherapy techniques is to visualize, localize, and segment the lesion for such focal therapy techniques. Verification of the correct position of the CTV and OAR on a daily basis during treatment is an issue of ongoing research and development in image-guided radiation therapy (IGRT) [9,10].

For the best delineation of these target volumes, multiparametric magnetic resonance imaging (mpMRI) represents the clinical gold standard. The annotated mpMRI maps are subsequently registered with computed tomography (CT) images for IGRT planning. In the clinical routine, this requires MR accessibility, which might not be available for all radiotherapy departments. However, CT scanners are standard imaging workhorses available in almost all radiotherapy departments.

Standard contrast-enhanced CT is, thus far, not able to visualize PCa sufficiently for computerized treatment plan optimization for focal PCa radiotherapy. To overcome this limitation, dynamic contrast-enhanced (DCE)-CT, which provides quantitative and qualitative information about the vascularization of the prostate, seems to improve the applicability of CT based on the initial feasibility studies [11–14]. The latest generation of CT scanners can provide DCE-CT examinations that offer high spatial and contrast resolution in combination with a moderate radiation dose. These scanners might facilitate the use of DCE-CT for the evaluation of prostate cancer and for microboost planning in patients in whom mpMRI is not possible.

Therefore, the aim of this study was to prospectively evaluate dynamic DCE-CT for the detection of PCa lesions using qualitative and quantitative image analysis compared to mpMRI of the prostate.

## Materials and methods

### Patients

This prospective study was approved by the ethical committee of the Medical University of Vienna (Nr. 1789/2015) and in accordance with the Declaration of Helsinki. From December 2015 to May 2018, consecutive patients without contraindications to MRI or CT of the prostate gland were eligible for this study. *A priori* exclusion criteria were CT contrast media allergic reaction history and renal insufficiency. All patients gave their written, informed consent prior to imaging.

All patients received an mpMRI within a three-month time window of the dynamic DCE-CT examination. Only patients with an MRI-proven suspicious lesion (≥PI-RADS 3) in the peripheral zone (PZ) who were scheduled for MRI-guided transrectal biopsy were further included in this study. Patients with a history of prostate therapy (e.g., brachytherapy) or therapy to other organs in the vicinity of the prostate, or hormonal therapy, were excluded.

The study cohort included 14 patients with PCa in the PZ. The mean age was 67 years (range from 57 to 78 years) and the mean PSA was 8.1 ng/ml 40 (range from 3.5 to 26.0 ng/ml). Patient characteristics are given in Table 1.

**Table 1:** Patient characteristics.

Parameter	Value
No. of patients	14
Mean age (y)	67 (57–78)
Mean PSA level (ng/mL)	8.1 (3.5–26)
Mean lesion diameter on MRI images (mm)	11 (7–24)
PI-RADS	
IV	11 (79%)
V	3 (21%)
Gleason score	
3+3	3 (21%)
3+4	6 (43%)
4+5	5 (36%)

PSA, prostate-specific antigen; PI-RADS, Prostate Imaging Reporting and Data System

### MRI

The mpMRI examinations were performed using a vendor-supplied, combined spine array coil and a body array receive-only coil on a 3T MRI system (Tim Trio, Siemens Healthineers, Erlangen, Germany). To avoid deformation of the prostate, no endorectal coil was used. The examination was performed in a feet-first supine position. To keep the level of peristaltic artefacts low, an anti-peristaltic agent (10mg hyoscine butyl-bromide, Buscopan^®^, Boehringer Ingelheim GmbH, Germany) was injected.

Patients underwent the following MP-MRI protocol:

- Anatomical T2w turbo spin echo in all three planes (field of view [FOV] 200mm; 20 slices at 3.0.mm; matrix 320).
- Axial echo planar imaging DWI sequence (Spectrally adiabatic inversion recovery [SPAIR] fatsat; matrix and FOV, 160 and 260 mm, respectively; slice thickness, 3.6 mm; 4 b-values [0, 100, 400, and 800 s/mm^2^];). Apparent diffusion coefficient maps (monoexponential calculation) were automatically constructed by the scanner software using DWI images of all b-values. In addition, high b-value images of 1400 s/mm^2^ were calculated as suggested in PI-RADS v2 [15].
- Contrast-enhanced (DCE) MRI was acquired using a view-sharing, three-dimensional, T1-weighted gradient echo sequence (TWIST) (70 repetitions; TWIST k-space subsampling with central region A 30% and sampling density 25%, resulting in a temporal resolution of 4.22 sec; FOV 260 mm; matrix 160). Gadoterate-meglumine (Gd-DOTA, Dotarem^®^, Guerbet, France) was injected intravenously after three baseline-scans as a bolus (0.2ml/kg body weight), using a power injector at a flow rate of 4ml/s, followed by a flush of 20ml of saline solution.

### Standard of reference

All lesions were verified by target-MRI in-bore biopsy, which was performed on a 1.5T MR unit (Magnetom Avanto fit, Siemens Healthineers, Erlangen, Germany) as described previously [16]. One radiologist with six years of experience performed the transrectal biopsy. Per lesion, at least three-to-five biopsy cores were obtained. A specialized urogenital pathologist analyzed the biopsy specimens according to recent guidelines [17].

### DCE-CT imaging protocol

CT examinations were performed on a 2 × 192-slice, dual-source CT system (Somatom Force, Siemens Healthineers, Erlangen, Germany). The DCE-CT was planned based on the *a priori* performed non-contrast pelvis CT with the following acquisition protocol: Active automatic tube current modulation (CARE Dose 4D; Siemens Healthineers Forchheim, Germany); 120 mAs reference tube current; 0.5 s rotation time; pitch 0.6; 192 × 0.6 mm detector collimation. After delineation of the prostate in the non-contrast scan, DCE-CT was performed using the following acquisition protocol: Depending on patient’s body weight, tube voltage was set to 80 or 90 kVp, rotation time was 0.25 s, and slice collimation 48 × 1.2 mm, with a slice width of 5 mm. The scan length was set to 114 mm. The perfusion scan consisted of 26 scans within a window of 47 seconds. The first 20 examinations were obtained at 1.5-second intervals, the next six at three-second intervals. The increase in intervals was selected to reduce the radiation exposure after the arterial influx. For the DCE-CT, all patients received an i.v. injection of a 70-mL bolus of nonionic iodinated contrast agent (5.0 mL/sec, Iomeron 400, Bracco Imaging, S.p.A., Milan, Italy) followed by a saline chaser of 40 mL at 5mL/sec using an automatic double-head injector. Patients were allowed to breathe normally during the perfusion examination. For the non-contrast scan, image reconstruction was performed using advanced modeled iterative reconstruction (ADMIRE, Siemens Healthineers, Erlangen, Germany) software at an IR strength level 3, which represents a standard level setting for non-contrast CT at our institution. DCE-CTs were post-processed on a SyngoVia workstation (syngo.via, version B20A; Siemens Healthineers; Forchheim; Germany) using the “Perfusion” application and the “Tumor” protocol with default settings. The software automatically generated a smoothed arterial time-enhancement curve and colorimetric maps of blood volume (BV), blood flow (BF), flow extraction production (FEP), and mean transit time (MTT), with each pixel representing a parameter value. Reconstructed images were imported into the local picture archiving and communication system. Dose-length-product (DLP) of the DCE-CT examinations and of the total CT examination consisting of the non-contrast pelvic CT scan and the DCE-CT scan were assessed.

### Subjective image quality of perfusion CT images

Two radiologists (Reader 1 = O1; Reader 2 = O2) (with four and six years of MRI and CT experience, respectively) blinded to the clinical history of the patients, independently assessed CT images with at least 14 days between the readings.

### Overall image quality

A score based on a LIKERT Scale ranging from 1 to 5 was used to describe the overall image quality of DCE-CT images:

1 = images non-diagnostic;
2 = images poor but still interpretable;
3 = image quality acceptable;
4 = image quality good;
5 = image quality excellent.

### Lesion visibility

Prostate lesions were subjected to qualitative analysis using visual grading analysis. The readers evaluated how accurately lesions could be delineated and assigned a confidence score ranging from 1–5. In patients with more than one lesion, an index lesion was assessed. The index lesion was determined as the largest and best-visible lesion. The ordinal score was defined as:

1 = non-diagnostic,
2 = poor but still interpretable,
3 = acceptable,
4 = good,
5 = excellent.

**Fig 1:**
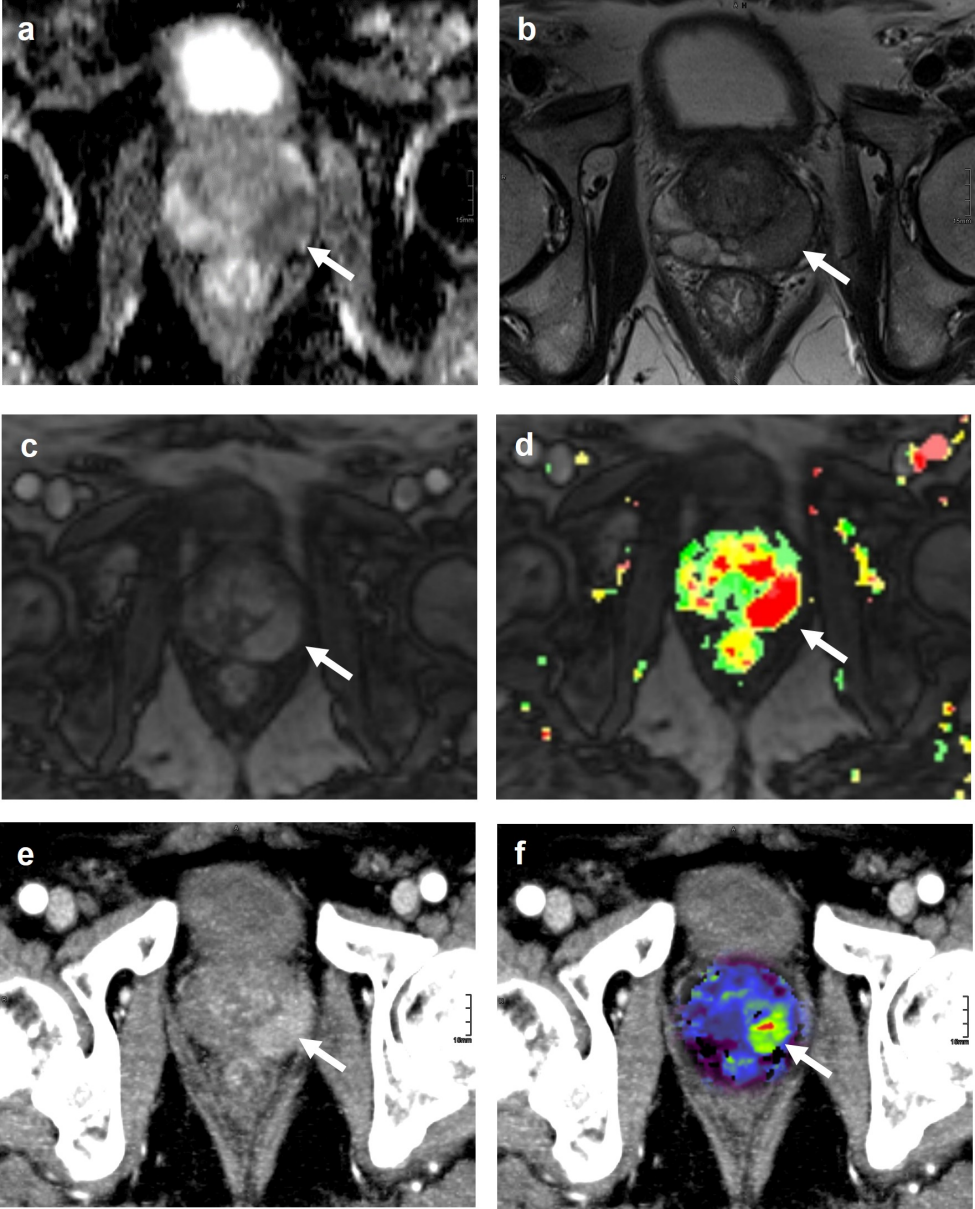
Examples of lesion delineation and image quality. Images were obtained in a 78-year-old, biopsy-naive man. DWI images (A), 2D T2w images (B), dynamic contrast-enhanced MRI (C), pseudocolor maps (D), CT-MIP images (E), and CT images with blood flow overlay (F), in the axial plane are shown. In the pseudocolor map, tissue is colored according to its contrast uptake over time. Green areas represent a continuous uptake over time. Orange areas represent tissue that shows a plateau phase, and red areas represent tissue with a washout. A lesion was detected in the left peripheral zone (arrow). For lesion delineation and image quality, the score was excellent. Histopathology obtained by MRI-guided biopsy confirmed a PCa Gleason score 7 (3+4).

**Fig 2:**
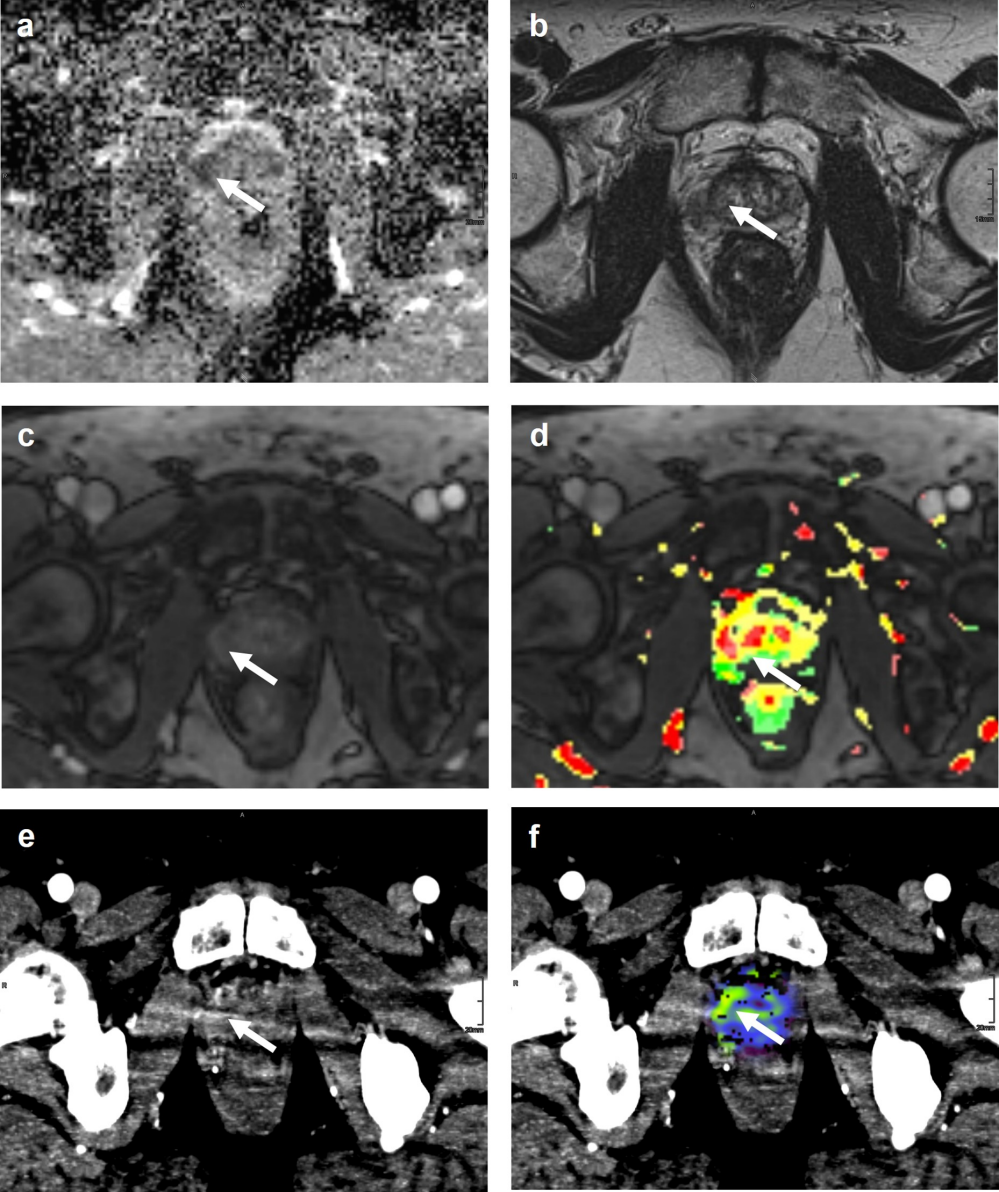
Examples of lesion delineation and image quality. Images were obtained in a 63-year-old, biopsy-naive man. DWI images (A), 2D T2w images (B), dynamic contrast-enhanced MRI (C), pseudocolor maps (D), CT-MIP images (E), and CT images with blood flow overlay (F), in the axial plane are shown. In the pseudocolor map, tissue is colored according to its contrast uptake over time. Green areas represent a continuous uptake over time. Orange areas represent tissue that shows a plateau phase, and red areas represent tissue with a washout. A lesion was detected in the right peripheral zone (arrow). For lesion delineation and image quality, the score was acceptable. Histopathology obtained by MRI-guided biopsy confirmed a PCa Gleason score 7 (4+3).

Examples of image quality and lesion visibility are provided in Fig 1 and Fig 2.

### Objective tumor perfusion parameter analysis

Quantitative image analyses were performed once by Reader 1. The volume of interest (VOI) covering the whole tumor was drawn on the maximum intensity projection (MIP) DCE-CT images. The MR images were used to identify the correct tumor location. Control VOIs were placed on the contralateral side in suspected healthy tissue. For every VOI, a mean enhancement curve was calculated and analyzed in the SyngoVia workflow.

### Statistical analysis

Comparisons between CT perfusion parameters within a patient were tested by a paired t-test. Ordinal visual analysis ratings were analyzed by visual grading characteristic (VGC) analysis, a non-parametric, rank-invariant statistical method derived from receiver operating characteristics (ROC) analysis, with the imaging method used as the classification and the visual grading used as the predictor variable [18]. VGC was used to evaluate the difference in image quality between the MIP reconstruction and CTP images. The area under the VGC curve (AUC) was deemed to be a measurement of the difference in image quality when comparing two or more modalities. The range value for the AUC is 0.5–1.0, whereas an AUC of 0.5 represents no superiority of a method with regard to the rated image quality criterion, and a value of 0 or 1.0 represents absolute inferiority or superiority of the respective method with regard to the evaluated image quality method. ROC analysis was used to compare the diagnostic performance of tumor delineation using MIP and CTP images.

All statistical analysis was performed using SPSS 24.0 (IBM Corp., Armonk, NY, USA). Intra-class correlation coefficient (ICC) estimates and their 95% confident intervals were calculated based on a mean-rating (k = 2), absolute-agreement, two-way mixed-effects model [19].

Data are expressed as mean ± standard deviation or median and range. A two-sided p-value <0.05 was considered significant.

## Results

### Prostate cancer detection rate

Cancer detection rates for the readers were 57% and 78% for DCE-CT and 92% for both readers for DCE-MRI. There were no significant differences in the DCE-CT detection rate between Gleason scores.

### Objective image parameters

**Fig 3:**
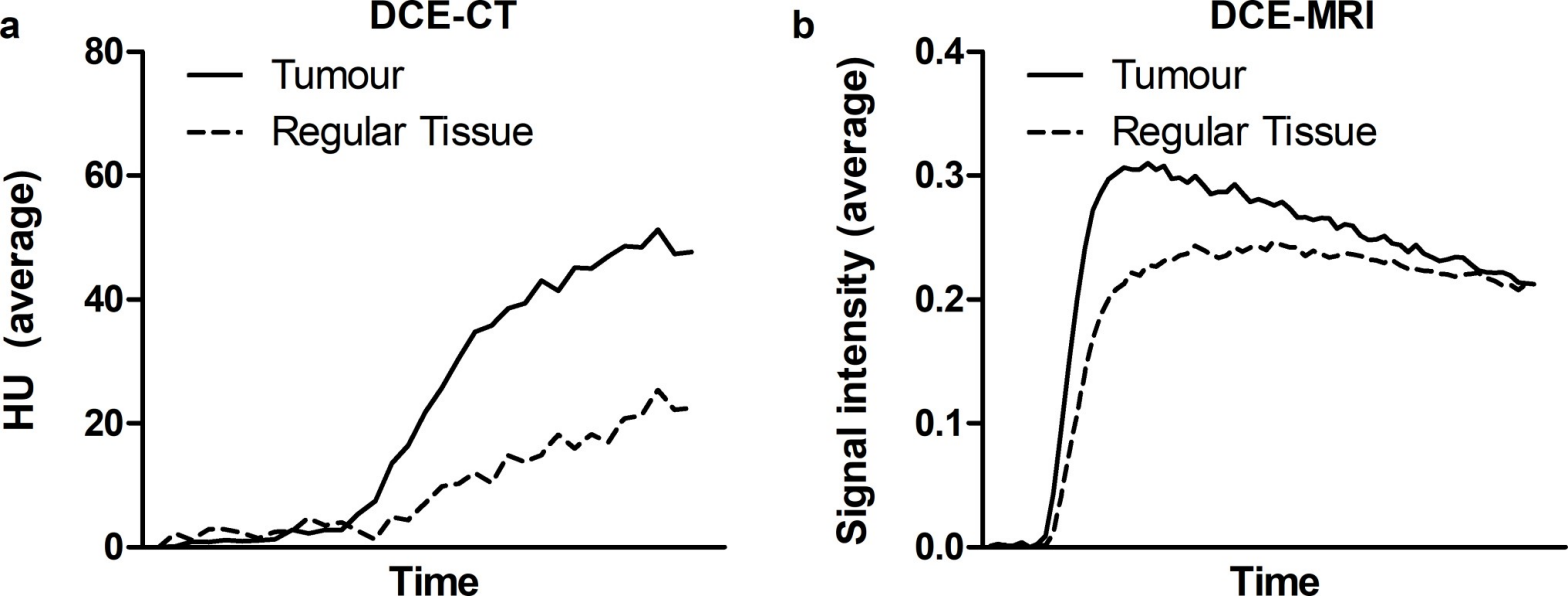
Average time curves of contrast uptake in PCa versus healthy regular prostate tissue. (A) Dynamic contrast-enhanced computed tomography (DCE-CT) and (B) dynamic contrast-enhanced magnetic resonance imaging (DCE-MRI), depicted for tumors in the peripheral zone. HU, Hounsfield Units.

Patients with PC showed significantly elevated DCE-CT perfusion parameters compared to regular prostate tissue (Table 2). In addition, a time-curve analysis showed an increased rapid uptake of the contrast agent in PCa in the PZ compared to regular prostate tissue both on the DCE-CT and DCE-MRI (Fig 3, p<0.001).

**Table 2:** DCE-CT perfusion parameters.

	MIP	BF	BV	MTT	FEP
Tumor	116.8 ± 22.8***	48.4 ± 23.7***	5.6 ± 5.6***	5.3 ± 1.5**	25.4 ± 14.5***
Regular	84.2 ± 16.4	21.6 ± 5.4	1.9 ± 1.7	4.2 ± 0.8	12.6 ± 5.9

MIP maximum intensity projection (HU), BF blood flow (mL/min/100g), BV blood volume (mL/100 mg), MTT mean transit time (seconds), FEP flow extraction product (mL/min/100g)

*p<0.05

**p<0.01

***p<0.001

### Subjective image parameters and lesion visibility

Of the 14 PCa patients, the median (range) lesion visibility scores from both readers were 3 (1–5) and 2.5 (1–5) for MIP reconstructions and CTP images, respectively. Examples of excellent or acceptable lesion delineation examinations are shown in Fig 1 and Fig 2. The median (range) image quality scores were 5 (3–5) for the MIP reconstructions and 4.5 (3–5) for the CTP images.

### Visual grading characteristics analysis

**Fig 4:**
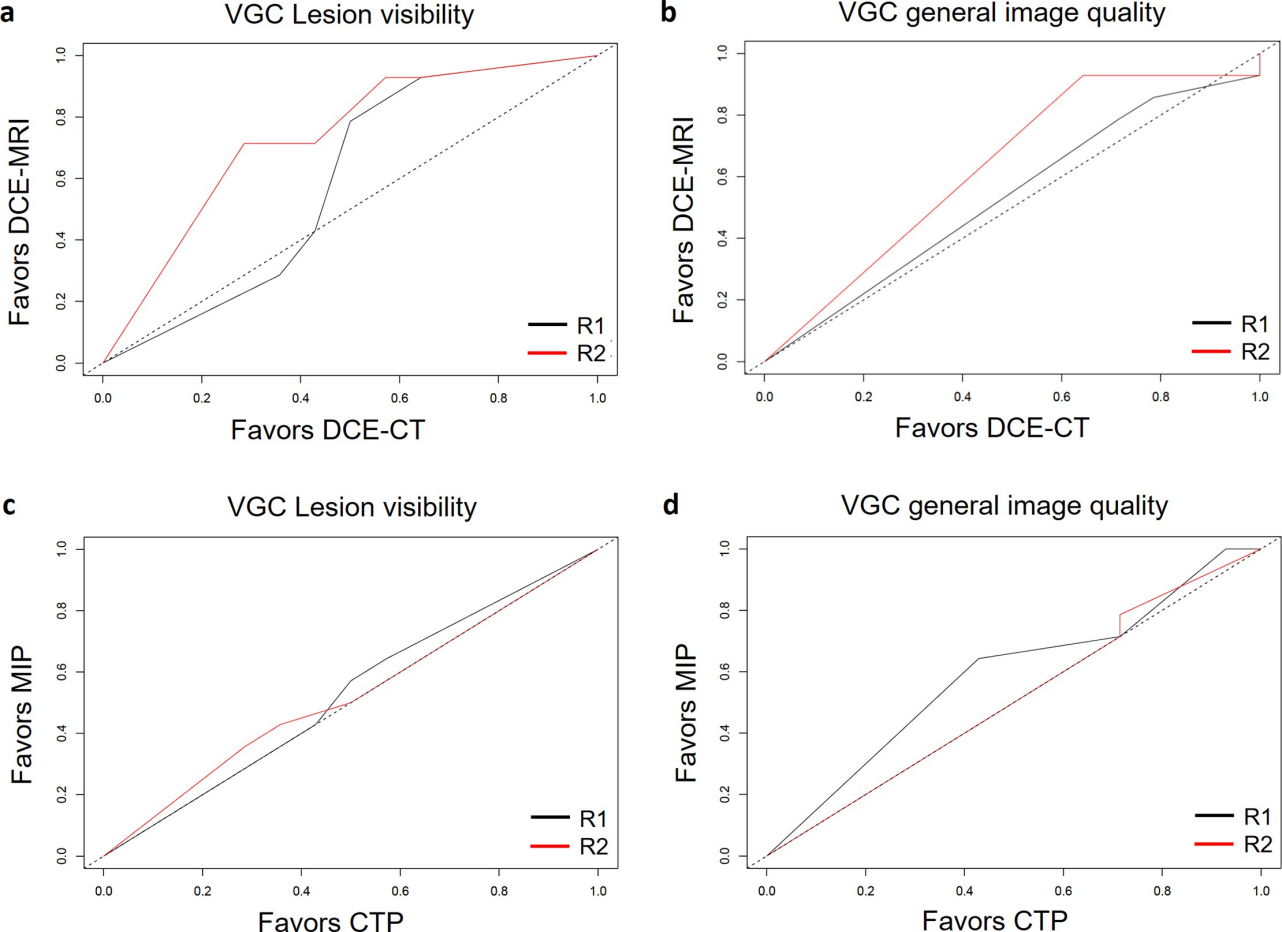
**Visual grading characteristic curves for lesion visibility (A, C) and image quality (B, D) assessment comparing DCE-CT and DCE-MRI (A, B) and DCE-CT MIP and CTP (C, D) reconstructions.** R2 scored DCE-MRI significantly better than DCE-CT in terms of lesion visibility (VGC 0.73, p = 0.01) (A). There were no significant differences in terms of general image quality between DCE-CT and DCE-MRI (B). There was no significant difference between the different image reconstructions with regard to lesion visibility and image quality (p>0.05) (C, D). R, reader; VGC, visual grading characteristics; DCE-CT, dynamic contrast-enhanced computed tomography; DCE-MRI, dynamic contrast-enhanced magnet resonance imaging; MIP, maximum intensity projections; CTP, computed tomography perfusion parameters.

For R1, the area under the VGC curve was significantly better for DCE-MRI compared to DCE-CT: R1: 0.53 (0.36–0.70), p = 0.01, while the VGC was not significantly different for R2: 0.73 (0.55–0.91), p = 0.44 (Fig 4A). The area under the VGC did not differ between DCE-CT and DCE-MRI reconstructions in terms of image quality: R1 0.53 (0.36–0.7), p = 0.72; R2 0.63 (0.47–0.78), p = 0.1. (Fig 4B). The area under the VGC curve did not differ between DCE-CT MIP reconstructions and CT color maps in terms of lesion visibility—R1 0.53 (0.31–0.73), p = 0.83; R2 0.52 (0.31–0.74), p = 0.85 (Fig 4C)—or image quality—R1 0.58 (0.39–0.79), p = 0.39; R2 0.51 (0.34–0.69), p = 0.91 (Fig 4D).

### Inter-reader agreement

There was an excellent inter-reader agreement for lesion delineation for MIP reconstructions, with an ICC 0.989 (0.964–0.996) and for CTP maps, with an ICC 0.988 (0.964–0.966) (for both p<0.001). In addition, there was a good agreement for MIP image quality, with an ICC 0.953 (0.857–0.985) and for CTP image quality, with an ICC 0.802 (0.378–0.937).

### Radiation dose

Mean DLP of the DCE study performed over the z-axis of the tumor was 1376.5 ± 168.1 mGy*cm, which corresponded to an effective dose of 23.4 ± 2.9 mSv, if using a conversion factor of 0.017 for the pelvis region [20]. Mean DLP of the total non-contrast pelvic CT was 153.9 ± 22.8 mGy*cm, which corresponded to an effective dose of 2.6 ± 0.4 mSv, resulting in an mean DLP for the whole CT examination of 1530.5 ± 182.7 mGy*cm, which corresponded to an effective dose of 26 ± 3.1 mSv.

## Discussion

In the present study, we investigated the feasibility of DCE-CT for the identification and delineation of target volumes in patients with PCa. We found that, in patients with PCa, DCE-CT of the prostate allows accurate identification and delineation of target volumes in the PZ. In particular, objective image parameters that included all lesions showed a significant difference between malignant and regular prostate tissue.

In the clinical scenario of patients with histology-proven PCa who are scheduled for focal boost therapy to the macroscopic tumor area, exact tumor structure/lesion delineation using a clinically suitable method is of utmost importance for treatment planning [5,21,22]. The excellent soft tissue contrast of MRI has been proven to accurately delineate PCa [23], while CT has been of limited value thus far in this respect. Due to technical improvements in CT hardware and post processing software, there is growing evidence that suggests that CT is able to detect abnormalities of the prostate gland. In a retrospectively collected series of pelvic CT in 27 PCa patients, the overall agreement for tumor detection between mpMRI and single-phase CT was 85.2% [24]. In another retrospective study, Glazer et al. reevaluated non-dynamic, contrast-enhanced CT of the abdomen and pelvis in 90 patients with PCa and 49 patients without PCa. A visual grading score of 5 increased the likelihood of a higher-grade PCa from 31% to 81% in this study population [25]. These two studies might suggest that, even in single phase pelvic CT, a PCa lesion can be detected. However, as contrast material arrival and inflow in the prostate gland varies depending on physiological conditions, such as heart rate and cardiac ejection volume, a dynamic acquisition might provide a more precise evaluation of healthy and malignant prostate tissue. The tumor detection rate using DCE-CT in our study ranged between 57% to 78%, and, therefore, was inferior to the referenced study above with 85.2%. These differences are mainly due to the applied selection criteria in the current study where only patients scheduled for MRI-guided prostate biopsy were included, with consequently smaller lesions with a lower Gleason score, compared to the retrospective studies. The relatively high difference in the detection rate between the two readers is most likely attributable to differences in experience level in assessing DCE-CT images. The agreement between readers, as well as general performance, will increase as the training levels increase with this new technique. Similar to Glazer et al. [25] and Jia et al. [24], we found that DCE-CT enables the detection and differentiation of PCa in the PZ. A veritable advantage of DCE-CT compared to single-phase CT is the ability to generate MIP images and perfusion maps. Whereas MIP images simply display the maximal attenuation obtained during the dynamic acquisition for each voxel, perfusion maps represent quantitative biological parameters, including BF, BV, MTT, and FEP.

As expected, PCa showed an early uptake of contrast agent, followed by a rapid wash-out, compared to healthy tissue. Consequently, PCa were visualized as hyper-attenuating lesions on MIP reconstruction with altered perfusion parameters compared to the benign prostate tissue, which is in line with previous investigations [14,26–28]. Interestingly, subjective image parameters for lesion visualization were rated better for the MIP reconstructions compared to the perfusion maps. In contrast to single-phase CT, the advantage of MIP reconstructions remains that they seem to be independent of variances in contrast material arrival. This might be due the fact that the maximum hyper-attenuating voxel is displayed, and thus, improves the visualization of hyper-attenuating lesions.

DCE-CT might be of great value for patients with PCa in a focal radiotherapy setting who are not suitable for MR due to contraindications, such as pacemakers, metal implants, or claustrophobia. DCE-CT might act as an alternative to MRI in these patients to localize the area of macroscopic tumor tissue and use this information for accurate RT planning. DCE-CT may also lead to reduced costs and time saved by not having to acquire an MRI. Although the radiation dose of the DCE-CT of approximately 26 mSv was high in this study, further studies could reduce this by lowering the number of scans and increasing the time gap between scans. However, the total added radiation dose is low compared to the dose delivered during radiotherapy [29].

In addition to lesion detection and lesion segmentation, quantitative and other advanced imaging methods have been explored for response assessment [2,30,31]. Although, at present, most studies concerning lesion characterization for both segmentation and response assessment focus on mpMRI, DCE-CT might also be of value in the response assessment and serve as an imaging biomarker for these patients.

This study has several limitations that need to be discussed. First, the total number of patients is low and only patients with PZ tumors were included. Second, as we included patients with Gleason scores 6–9, we had a high number of patients with low-Gleason-score tumors. Based on previous reports, in which higher-grade tumors were better visible on pelvic CTs [14,25], it is likely that a higher number of high-grade tumors would have improved the detection rate of CTs. In addition, we did not calculate the false-positive or false-negative rate. We believe that our study design was not suited to accurately calculate these metrics, because we included only patients with prostate carcinoma in the peripheral zone. Finally, we did not include patients with gold markers/fiducials in place. These might reduce the DCE-CT image quality.

## Conclusion

In conclusion, our preliminary results suggest that, in patients with PCa, DCE-CT allows identification and visualization of macroscopic tumor areas in the peripheral zone in up to approximately 80 percent of patients. Further studies are needed to evaluate the potential of DCE-CT as a non-invasive imaging biomarker for treatment response assessment.

## Abbreviations

PCa: prostate cancer
PZ: peripheral zone
VGC: visual grading characteristics
AUC: area under the VGC curve
DCE-CT: dynamic contrast-enhanced computed tomography
MIP: maximum intensity projections
CTP: computed tomography perfusion
IMRT: intensity-modulated radiation therapy
IGRT: image-guided radiation therapy
ICC: intra-class correlation coefficient
BV: blood volume
BF: blood flow
FEP: flow extraction production
MTT: mean transit time
